# Relationships between Linear Sprint, Lower-Body Power Output and Change of Direction Performance in Elite Soccer Players

**DOI:** 10.3390/ijerph17176119

**Published:** 2020-08-22

**Authors:** Monika Papla, Michal Krzysztofik, Grzegorz Wojdala, Robert Roczniok, Marcin Oslizlo, Artur Golas

**Affiliations:** Institute of Sport Sciences, The Jerzy Kukuczka Academy of Physical Education in Katowice, 40-065 Katowice, Mikolowska 72a, Poland; m.papla15@gmail.com (M.P.); wojdala.grzegorz@gmail.com (G.W.); r.roczniok@awf.katowice.pl (R.R.); m.oslizlo@awf.katowice.pl (M.O.); a.golas@awf.katowice.pl (A.G.)

**Keywords:** agility, COD deficit, squat power, team sports, speed

## Abstract

The aim of this study was to investigate the relationship between linear sprint, power output obtained during a squat and change of direction (COD) performance. Fifteen elite soccer players participated in this study (age = 21.7 ± 0.72 years, body mass = 74.9 ± 9.11 kg, body height = 180.4 ± 7 cm, training experience = 9 ± 1.5 years). To examine these correlations a following battery of tests were carried out: 20-m linear sprint, one-repetition maximum (1RM) squat strength, peak power output obtained during a squat at 50% 1RM and time obtained in two 20-m COD tests with different angles of direction change (90° and 135°). In addition, COD deficits (90°-COD_DEF_ and 135°-COD_DEF_) for both COD tests were calculated. The Spearman’s rank order correlation showed a nearly perfect statistical relationship between the 90°-COD and the 90°-COD_DEF_ (r = 0.9; *p* < 0.001). In the case of 90°-COD_DEF_, there was a large statistical relationship with 135°-COD_DEF_ (r = 0.59; *p* = 0.021). Moreover, there was a nearly perfect statistical relationship between 135°-COD and 135°-COD_DEF_ (r = 0.91; *p* < 0.001). The statistically insignificant (*p* > 0.05) relationship between 20-m linear sprint time, power output obtained during a squat at 50% 1RM, 1RM squat strength level and both COD test, as well as both COD deficits were found. Results of the present study showed that 20-m linear sprinting speed, 1RM squat strength, power output obtained during squat at 50% 1RM and COD ability at 90° and 135° angles, are separate physical qualities. Moreover, it seems that COD deficit provides a more isolated measure of COD ability than the COD tests alone and does not must be limited to a specific angle, but provides knowledge about the COD ability in a range of other angles, at least concerning 90° and 135° COD angles.

## 1. Introduction

Most team sports require players to jump, sprint and change direction very frequently; such sports include American football, rugby and soccer [1,2]. During a soccer match, players make over 700 turns with different changes of direction (COD) [3], as well as numerous jumps and sprints [4]. During a COD, athletes’ ability to accelerate, decelerate and reaccelerate in a new direction requires a rapid application of force. The acceleration phase in COD and linear sprint involves similar technical factors, so improving acceleration capability may be beneficial in terms of accelerating quickly after successive COD maneuvers and transition between them. Hence, it can be assumed that linear speed and lower-body power output may affect COD performance. Given that, many researchers examined the relationship between the performance of the above mentioned high-intensity actions and reached varying degrees of association, further studies seem justified [5,6,7,8,9,10]. Previous studies indicate a statistically significant large to very large relationships between linear sprints and different COD sprints (45°, 60°, 90° and 135° changes of direction) at various distances [7,8,9,11]. Additionally, Suarez-Arrones et al. [10] showed a statistically significant moderate and large relationship between 10-m linear sprint and equal distance different COD sprints (90° and 180°; respectively). At the same time, Loturco et al. [9] revealed no statistically significant relationship between 100° COD test and linear sprints at 5 m, as well as on 10 m.

Profiling of COD performance is difficult due to a variety of tests used in research. Certain COD tests differing in length, angle and a number of direction changes. Therefore, they may have different physical and mechanical requirements. In regards to the COD angles, as suggested by Falch et al. [12] and Bourgeuis et al. [13] angels below 90° are more velocity-oriented in contrast to the angles above 90°, which are more force-oriented. Consequently, it can be assumed that COD performance with the angles below 90° and that which exceeding 90° should be measured and trained separately. Regarding the number of direction change, a majority of COD tests consists of two or more turns [14], however in case of soccer players, it is rare to change direction more than three times during matches [15]. With respect to the fatigue caused by hundreds of COD during a soccer match, the use of COD tests with several or repeated COD tests seems warranted when the COD ability is assessed among soccer players.

The solution that can shed new light on the assessment of COD ability, is a measurement of COD deficit. However, to date little attention has been given to the relationship between COD deficit and the performance of the high-intensity actions and COD tests [2,10,15,16]. The COD deficit is an additional time that athletes need, to complete running with a COD in comparison to straight-line sprint at the same distance (e.g., athlete’s 20-m sprint time is subtracted from the 20-m COD time). This difference in time allows to better isolate and identify an athlete’s ability to change direction [2]. The lower the value, the greater the COD ability. A study by Nimphius et al. [2] showed a statistically significant positive relationship between COD deficit (measured as the difference between 10-m sprint time and 180° COD test) and COD test (505), but not with 10- and 30-m sprint time in male cricketers. In contrast, Loturco et al. [15] did not find a statistically significant relationship between COD deficit (measured as the difference between 20-m linear fly sprint and 100° COD test) and 100° COD test. However, the authors indicated a statistically significant positive moderate relationship between COD deficit and relative mean propulsive power obtained in the half-squat and sprint velocity at five meters. Furthermore, a large and nearly perfect relationship was found between 10- and 20-m linear flying sprint and COD deficit among elite soccer players. Moreover, Loturco and colleagues [15] found statistically significant positive large and nearly perfect relationship with 10-m and 20-m linear flying sprint, respectively. In reference to the Nimphius et al. [2], the COD deficit may be specific to the angle and, as shown by the results of previous studies, may also for the length of run, discipline and the type of start (standing vs. flying). Therefore, in order to increase the versatility of this measure, there is a need for research which compare the COD tests and COD deficits with different direction change and running length in the same group of athletes.

Therefore, the aims of this study were to examine the relationships between a 20-m linear sprint, 1RM squat strength, peak power output obtained during a squat at 50% 1RM, 90° and 135° COD tests. A second aim of this study was to analyze the possible relationships between the COD deficits (using 90° and 135° COD time and 20-m sprint time) with all considered tests. It was hypothesized that significant relationships between all measured variables will exist. The second hypothesis was that peak power output obtained in the squat and 20-m linear sprint would be positively correlated with the COD deficit and the magnitude of correlation will depend on the angle of change.

## 2. Materials and Methods

### 2.1. Experimental Design

To examine the relationship between linear sprint, lower-body maximum strength and power output as well as COD performance, the following tests were carried out: 20-m linear sprint, one-repetition maximum (1RM) squat strength, peak power output obtained during a squat at 50%1RM and two separate 20-m COD sprints, first with a 90° and the second with a 135° direction change angles. To assess a more isolated measure of the COD performance, the COD deficits for both conducted tests were calculated. Measurements were conducted on two different sessions, 72 h apart. Linear and COD sprints were performed during the first session, and 72 h later the lower-body power output was assessed.

### 2.2. Study Participants

Fifteen elite male soccer players from a professional team participated in the study (Second Polish League) (age = 21.7 ± 0.72 years, body mass = 74.9 ± 9.11 kg, body height = 180.4 ± 7 cm, training experience = 9 ± 1.5 years, 1RM squat = 200 ± 8.7 kg). The athletes were all full-time professionals who trained daily. All athletes had valid medical examinations and showed no contraindications to participate in physical fitness tests. The experimental sessions took place at the beginning of the pre-season. The athletes were instructed to maintain their normal dietary habits over the course of the study and not to use any supplements or stimulants for the duration of the experiment. Moreover, they were informed verbally and in writing about the experimental protocol, the possible risks and benefits of the study and the possibility to withdraw at any stage of the experiment. All players gave their written consent for participation. The study protocol was approved by the Bioethics Committee for Scientific Research (10/2018), at the Academy of Physical Education in Katowice, Poland and performed according to the ethical standards of the Declaration of Helsinki, 2013.

### 2.3. Testing Procedures

One week prior to starting the experimental sessions, all athletes were familiarized with the testing procedures and 1RM test for the Keiser Squat exercise. The experimental sessions were carried out at the same time of the day (between 9:00 and 11:00 a.m.) 72 h apart. Both sessions were preceded by the same warm-up protocol, which included 5 min of jogging, dynamic stretching, a single attempt of a 20-m linear sprint and two different COD sprints (90° and 135°) at submaximal intensity and 2 sets of body-weight squats. During the first experimental session, the athletes performed a 20-m linear sprint and 20-m COD sprints with two different turn angles (90° and 135°). All sprint tests were performed on an indoor court. The running times were recorded by two pairs of dual-beam Witty Gate photocells (Microgate, Bolzano, Italy) with the measuring precision of 0.01 s. The intraclass correlation coefficient for the test–retest reliability in times of linear sprinting and COD tests measured by used photocells ranged from 0.96 to 0.99. In the second experimental session peak power output obtained during a squat exercise at 50% 1RM was assessed using the Keiser Air 300 Squat pneumatic machine (Keiser Corporation, Fresno, CA, USA). This value of the external load was chosen because it is the lower value of the range that was indicated as optimal for obtaining the highest values of peak power outputs during a squat exercise [17]. The Keiser pneumatic resistance system utilizes air-pressurized resistance to maximize safety and allows for precision loading within one kilogram. The intraclass correlation coefficient for the test–retest reliability in the peak power output during a squat exercise measured by Keiser Air 300 Squat pneumatic machine was 0.97.

#### 2.3.1. One-Repetition Maximum Test

One week before the first experimental session the 1RM squat exercise test was performed on the Keiser Squat air pneumatic machine. After a standardized warm-up, the athletes performed 10, 6, 4 and 3 repetitions, starting at a load of 20 kg and progressing to 60–80% of their estimated 1RM. The first testing load was set to an estimated 90% 1RM and was increased by 5–10 kg for each subsequent attempt until the athlete was unable to perform a proper lift with a correct technique. The 1RM test result was determined within 5 attempts, with 5 min of rest in between attempts. All testing was performed with a constant tempo of movement [18,19]. The strength coaches were present throughout the procedure of 1RM testing. The athletes started from an upright position, with the knees and hips fully extended, the stance approximately shoulder-width apart with both feet positioned flat on the floor in parallel or externally rotated to a maximum of 15° [20], hands were placed on the hand grips, and this setting was carefully replicated on every lift. From this position, they were required to descend until contact with the bench (without losing muscle tension) and then perform the concentric phase of the movement in an explosive manner. The height of the bench allowed each athlete to descend with the hips below the knee line to keep constant squat depth. No weight-lifting belts, shoes or other supportive garments were permitted.

#### 2.3.2. Linear Sprint Test

Following the warm-up, all athletes performed 2 maximal 20-m linear sprints, interspersed with 5 min rest intervals. The athletes started with the front foot placed 0.5 m behind the first timing gate, to prevent any early triggering of the start gate. The athletes started when ready to eliminate the reaction time effect. The fastest time from both attempts was retained for further analysis.

#### 2.3.3. Change of Direction Tests

**Figure 1:**
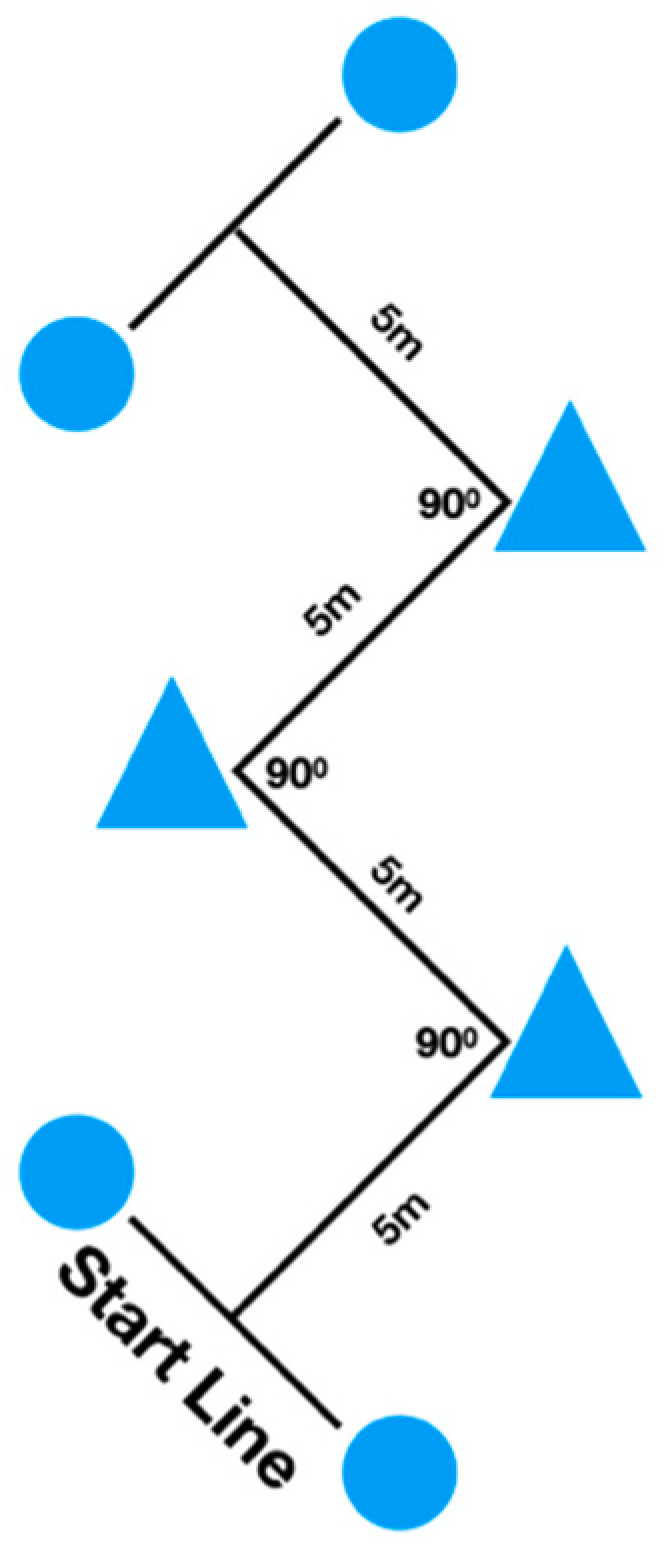
Schematic presentation of the 90° change of direction test. Circles represent the positions of the photocells.

**Figure 2:**
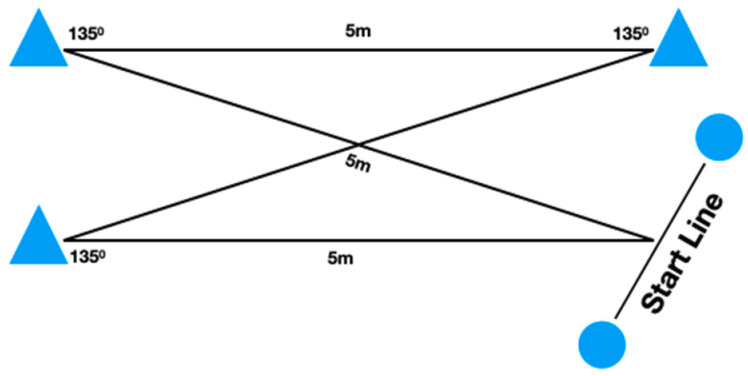
Schematic presentation of the 135° change of direction test. Circles represent the positions of the photocells.

Following the 20-m linear sprint test, the participants were provided with a 5 min rest interval before completing the COD tests. The two COD tests consisted of four 5-m sections marked with cones set at 90° (90°-COD) and 135° (135°-COD), requiring the athletes to decelerate and accelerate as fast as possible around each cone (Figure 1 and Figure 2). The players executed two attempts of each COD test with 5 min rest intervals in between attempts and tests. The fastest time from each COD test was retained for further analysis.

#### 2.3.4. Change of Direction Deficits

To provide a measure of each athletes’ COD ability, an adapted COD deficit calculation was used. The COD deficit for both angles (90°-COD_DEF_ and 135°-COD_DEF_) was calculated as follows: 20-m linear sprint time −90°-COD for 90°-COD_DEF_ and 20-m linear sprint time −135°-COD for 135°-COD_DEF._

#### 2.3.5. Lower-Body Power Output

Following the warm-up, a lower-body power output test was assessed using the squat exercise performed on the Keiser Squat air pneumatic machine at 50% 1RM. The strength coaches were present throughout the test to ensure safety and to carefully replicate the setting on each lift. All athletes were instructed to perform the concentric phase of the movement as fast as possible. As during the 1RM test, no weight-lifting supportive garments were permitted. The players executed two attempts of lower-body power output with 5 min rest intervals in between. The best peak power output was considered for further analysis.

### 2.4. Statistical Analysis

All statistical analyses were performed using Statistica 9.1 (Hillview, Palo Alto, CA, USA). The physical tests of this study were: 20-m linear sprint, 1RM squat strength, peak power output in squat, 90° and 135°-COD tests and 90° and 135°-COD_DEF_. Data are presented as means and standard deviations (SD) with 95% confidence intervals. The normality of the data were examined by the Shapiro–Wilk test. Due to the lack of normal distribution of the studied variables (linear sprint and 135°-COD), the Spearman’s rank order correlation was used to determine the relationship between all conducted tests. Correlations were evaluated as follows: trivial (0.0–0.09), small (0.10–0.29), moderate (0.30–0.49), large (0.50–0.69), very large (0.70–0.89), nearly perfect (0.90–0.99) and perfect (1.0) [21]. The significance level for the correlation analysis was set as *p* < 0.05.

## 3. Results

Descriptive data for all the tests is shown in Table 1, while the Spearman’s rank order correlations between all measured variables are presented in Table 2. There were no statistically significant relationships between peak power output obtained during squat at 50% 1RM and all of the other measured variables. Further, there were no statistically significant relationships between squat 1RM and all of the other measured variables. Similarly, no statistically significant relationships between linear sprint and all of the other measured variables were found. In regards to the 90°-COD there was a nearly perfect statistically significant relationship with 90°-COD_DEF_ (r = 0.9; *p* < 0.001). In case of 90°-COD_DEF_, there was a large statistically significant relationship with 135°-COD_DEF_ (r = 0.59; *p* = 0.021). Moreover, there was a nearly perfect statistically significant relationship between 135°-COD and 135°-COD_DEF_ (r = 0.91; *p* < 0.001).

## 4. Discussion

The main finding of this study was that 1RM squat strength, peak power output obtained during a squat at 50% 1RM and the 20-m linear sprint was not significantly correlated with each other and with any of the measured COD tests. However, there was a nearly perfect statistical relationship between the 90°-COD and 90°-COD_DEF_ as well as between 135°-COD and 135°-COD_DEF_. Furthermore, there was a large statistical relationship between 90°-COD_DEF_ and 135°-COD_DEF_. The results indicated that 20-m linear sprinting speed, 1RM squat strength and power output obtained during squat at 50% 1RM, as well as COD ability at 90° and 135° angles, are separate physical qualities. Moreover, it seems that the COD deficit does not must be limited to a specific angle but provides knowledge about the COD ability in a range of other angles, at least concerning 90° and 135° COD angles. Additionally, these data suggests that the COD deficit provides a more isolated measure of COD ability than the COD tests alone due to the reduced effect of linear sprinting speed within a COD test among elite soccer players.

A wide range of studies has been conducted to determine the applicability of a given exercise that leads to enhancement of specific athletic performance [22,23,24,25]. However, various resistance exercises are significantly related to performance of selected physical fitness tests. The lack of a statistically significant relationship between, 1RM squat strength and peak power output obtained in the squat exercise at 50% 1RM and 20-m linear sprint, as well as with COD performance may be explained by different mechanical demands of the hip and knee extensors executing these high-intensity actions. A study by Contreras et al. [24] found that 6-week hip thrust training could be more beneficial in improving 10- and 20-m linear sprint times compared with front squat training, which may be superior in vertical jump height enhancement. In addition, González-García et al. [25] revealed that strength improvement after 7-week hip thrust training showed greater improvements in 10- and 20-m sprint, as well as in COD test (90°) in comparison to back squat training [25]. It is possible that the hip thrust has a stronger transfer to sprint running, whereas the squat has more influence on vertical jump performance [24]. Furthermore, a lack of relationship between 20-m linear sprint time and peak power output obtained in the squat at 50% 1RM may be related to the used external load and measured variable. In the current study, the assessment of correlations is based only on a single value of external load (50% 1RM; 100 ± 4.35 kg) and on peak power output. In turn, a study by López-Segovia et al. [26] showed significant positive correlations with 10-, 20- and 30-m linear sprint time with mean power output obtained in a full squat, but not with peak power output among soccer players. That relationship was found only at 70 kg which was close to the body mass of participants (93%), but not at lower loads (from 20 to 60 kg with 10 kg increments). While in the present study, the external load used during the squat significantly exceeded the body mass of participants (~133%). These findings partially confirm the suggestion of López-Segovia et al. [26] that certain levels of neuromuscular activation, assessed by mean power output generated at loads to body may be related with linear sprint performance. Thus, future studies should examine the relationship between hip thrust and squat exercises at a wide range of external loads versus linear sprint, as well as COD performance.

To the best of the authors’ knowledge, a limited number of studies have analyzed the relationship between linear sprint and the COD deficit among soccer players [15,27,28]. Findings of the current study revealed that 20-m linear sprint time was not statistically significant related to both COD deficits, what is contradict to findings of Loturco et al. [15]. The authors showed a statistically significant nearly perfect positive relationship between 100° COD deficit and 20-m flying start linear sprint in team-sport athletes. Moreover, Loturco et al. [15] found a statistically significant large positive relationship between 100° COD deficit and 10-m flying start linear sprint. Unfortunately, Loturco and colleagues [16] did not assess whether these results would be similar in the case of a linear sprint from the standing start. At the same time, Loturco et al. [28] revealed differences in the COD deficit between elite soccer players versus handball, rugby and futsal players. The COD deficit was significantly higher in soccer players in comparison with the remaining disciplines, which could be explained by the nature of these sports. It is important to note, that most COD-runs occur with angles between 0 and 90° (approximately 84%), with the next most common range of 90–180° (approximately 13%) in soccer players [3]. Therefore, the use of angles greater than 90° in COD tests should be considered with logical validity when the goal is to assess the relationships between high-intensity actions and COD performance among soccer players. However, angles greater than 90° occur much less frequently in a match, this should not be ignored, and players must also be prepared for such maneuvers. Therefore, the assessment of the relationship between COD tests and COD deficits with different angles, also exceeding 90°, seems justified from the training practice point of view. In the current investigation, there was a nearly perfect statistical relationship between performances in the COD tests and COD deficits for the same angles (90°-COD and 90°-COD_DEF_; 135°-COD and 135°-COD_DEF_). These results are in line with previous findings obtained by Nimphius et al. [2]. The authors revealed a large statistically significant relationship between the COD deficit and COD test time (505 test). Furthermore, there was a large statistically significant relationship between COD deficits between angles (90°-COD_DEF_ vs. 135°-COD_DEF_), while it was not the case for COD test (90°-COD vs. 135°-COD). The rationale for that results may be similar mechanical requirement between COD angles suggested by the Falch et al. [12] and Bourgeuis et al. [13], that angles below 90° are more velocity-oriented, while angles above 90° are more force-oriented. These data provides further support for the use of a COD deficit to evaluate an athlete’s COD ability, as it removes the influence of linear running speed on such tests. Therefore, it seems that the obtained relationship between the COD deficits indicates that this measure provides information about the athlete’s COD ability regardless of the angle, at least for 90°- and 135°-COD. Thus, the COD deficits measure provides valuable information for the coaches, which allows the preparation of an individualized training program, e.g., whether the athletes require a complementary program aimed at improving the COD ability.

The present study has some limitations which must be addressed. The first limitation of the study is the assessment of relationships based only on a single value of external load during a squat exercise (50% 1RM). Hence, the results of the presented study do not translate to other loads. Moreover, only a single running length (20 m) for linear sprint and COD tests was examined, as well as the same number of direction changes (3 turns). In addition, these running tests were performed during one experimental session, so the impact of fatigue on the result cannot be ruled out. Taking into consideration the nature of soccer, relationships between high-intensity actions and angles below 90° during COD should be analyzed, however sharper angles should not be completely ignored. Thus, future studies should provide a detailed relationship between different exercises (e.g., hip thrust) at a wide range of external loads and COD performance with variety of angles and numbers of direction change, in a large number of soccer players.

## 5. Conclusions

The results of the present study showed that 20-m linear sprinting speed, 1RM squat strength, power output obtained during squat at 50% 1RM and COD ability at 90° and 135° angles, are separate physical qualities. Moreover, it seems that COD deficit provides a more isolated measure of COD ability than the COD tests alone and does not must be limited to a specific angle, but provides knowledge about the COD ability in a range of other angles, at least concerning 90° and 135° COD angles. Thus, the lower the deficit time in athletes, the more effective the COD or the higher the ability of an athlete to COD relative to their physical ability for linear speed. What indicates that COD deficits provides an isolated measure of athletes’ COD ability and is not biased towards superior lower-body peak power output or 1RM squat strength level and 20-m linear sprint. Therefore, practitioners are recommended to evaluate COD performance based on COD deficits to detect the athlete’s capacity to change direction.

## 6. Practical Applications

The findings from this study show that COD deficit is an easy way to evaluate the COD ability in athletes. Therefore, coaches should use the COD deficit to enable the prescription of more targeted and individualized training programs to enhance the athletes’ capability to perform directional changes. Based on this, coaches can implement a more comprehensive training strategy depending on whether the athlete would benefit more from developing the ability to change direction or from improving linear sprint performance.

## Figures and Tables

**Table 1:** Descriptive data for all measured tests.

Test	Mean ± SD	95% CI
Squat 1RM (kg)	200 ± 8.7	192 to 208
Linear Sprint (s)	3.02 ± 0.1	2.97 to 3.08
90°-COD (s)	7.07 ± 0.25	6.93 to 7.2
135°-COD (s)	7.25 ± 0.28	7.09 to 7.4
90°-COD_DEF_ (s)	4.04 ± 0.21	3.93 to 4.16
135°-COD_DEF_ (s)	4.22 ± 0.24	4.09 to 4.35
Power Output Squat (W)	1408 ± 129	1337 to 1480

Mean ± standard deviation (SD); CI—confidence intervals; 1RM—one-repetition maximum; COD—change of direction; COD_DEF_—change of direction deficit.

**Table 2:** Spearman’s rank order correlations between all measured variables.

Test		Linear Sprint	Power Output Squat	90°-COD	90°-COD_DEF_	135°-COD	135°-COD_DEF_	Squat 1RM
Linear Sprint	r	/						
Power Output Squat	r	−0.30	/					
90°-COD	r	0.43	0.09	/				
90°-COD_DEF_	r	0.15	0.07	0.90 **	/			
135°-COD	r	0.29	−0.30	0.33	0.38	/		
135°-COD_DEF_	r	0.05	−0.27	0.42	0.59 *	0.91 **	/	
Squat 1RM	r	−0.16	−0.23	−0.17	−0.02	−0.05	−0.01	/

* statistically significant differences *p* < 0.05; ** statistically significant differences *p* < 0.01; r—Spearman’s rank order correlation; COD—change of direction; COD_DEF_—change of direction deficit.
